# Lymphomatoid Granulomatosis in a Patient with Chronic Lymphocytic Leukemia and Rapidly Progressing Peribronchovascular Pulmonary Infiltrates

**DOI:** 10.1155/2019/9870494

**Published:** 2019-01-21

**Authors:** Shahab Rezvani, Marie Tominna, Sayf Al-Katib, Marc D. Smith, Craig Cousineau, Ayad Al-Katib

**Affiliations:** ^1^Beaumont Health, Oakland University William Beaumont School of Medicine, Department of Diagnostic Radiology and Molecular Imaging, 3601 W 13 Mile Rd, Royal Oak, MI 48073, USA; ^2^Beaumont Health, Oakland University William Beaumont School of Medicine, Department of Clinical Pathology, 3601 W 13 Mile Rd, Royal Oak, MI 48073, USA; ^3^Wayne State University School of Medicine, Lymphoma Research Laboratory, 540 E Canfield Room No. 8829, Detroit, MI 48202, USA

## Abstract

Lymphomatoid granulomatosis (LG) is an EBV-associated angiodestructive lymphoproliferative disease with multiorgan involvement that predominantly affects the lungs. We present a case of a 72-year-old man with a history of chronic lymphocytic leukemia who presented with upper respiratory symptoms and multiple erythematous skin papules. Chest CT showed ill-defined, irregular solid pulmonary nodules with peripheral ground-glass opacities in a peribronchovascular distribution. The differential for this pattern of lung disease is vast which includes but is not limited to infection, vasculitis, sarcoidosis, lymphoma, and Kaposi sarcoma. Subsequent PET/CT showed rapid progression of lung opacities and marked FDG uptake of pulmonary opacities and skin nodules, which raised the question of Richter syndrome. Wedge biopsy under video-assisted thoracoscopic surgery was performed. Pathology showed an extensive lymphoid infiltrate involving lymphatic and bronchovascular bundles and consisting of a mixture of large lymphocytes and inflammatory cells. Special stains showed that the large lymphocytes expressed B-cell markers and EBV virus. Overall, the findings were consistent with LG.

## 1. Case

**Figure 1:**
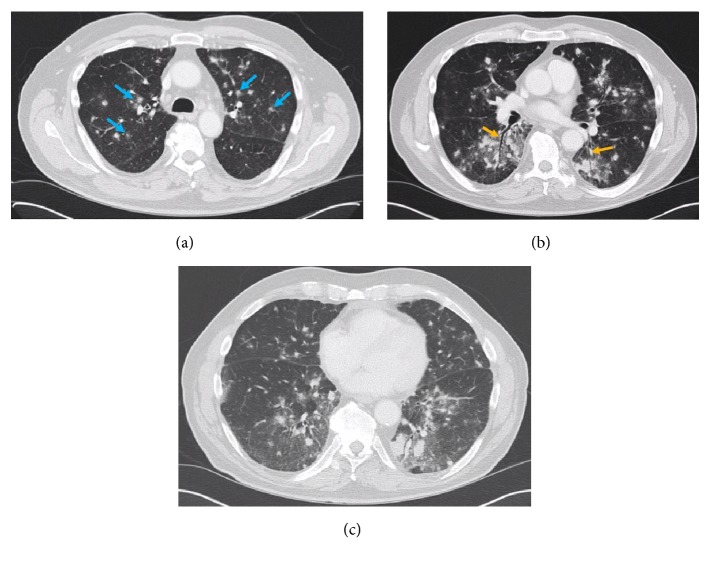
Axial lung window CT slices (a, b, c) at different levels demonstrate innumerable ill-defined solid nodules with peripheral ground-glass halos in a peribronchovascular distribution (blue arrows) and more prominent in the left lung. Air-bronchograms were seen (orange arrows).

A 72-year-old male with a long standing history of chronic lymphocytic leukemia (CLL) presented with upper respiratory symptoms including mild productive cough and dyspnea. An outpatient chest CT showed innumerable bilateral ill-defined solid pulmonary nodules in a peribronchovascular distribution, which were new from a prior scan 6 months earlier (Figure 1). Many of the lesions had a peripheral ground-glass halo. Nonenlarged mediastinal and bilateral axillary lymph nodes were suspected to be related to the patient's history of CLL. The pulmonary nodules were not a typical manifestation of CLL and other etiologies were considered such as atypical pulmonary infection, sarcoidosis, Kaposi sarcoma, and metastasis, even though patient had no other known malignancy. He was treated with antibiotics and steroids for his symptoms; however there was progressive clinical decline over several weeks and thus the patient was admitted for further work-up.

At the time of admission, vital signs showed exertional hypoxia, mild tachycardia in the low 100's, and a fever up to 102.4, which raised the concern for an infection and septic emboli. However, there were no significant pulmonary findings on exam. Skin examination revealed erythematous/purple skin papules on both lower extremities which broadened the differential to also include autoimmune and vascular etiologies.

Aside from normocytic anemia (Hb 11.6 mg/dL), initial laboratory evaluation with CBC and BMP showed no significant abnormalities. IgG levels were low. Blood and sputum cultures were negative. QuantiFERON was negative for tuberculosis. Serologies for aspergillus, blastomycosis, coccidioides, cryptococcus, histoplasma, HIV, and toxoplasma were negative. Bronchoalveolar lavage was negative for acid-fast bacilli, fungal organisms, and pneumocystis.

Immunologic evaluation was negative for ANCA, proteinase 3, and myeloperoxidase antibodies. This excluded granulomatosis with polyangiitis as a differential diagnosis. Bronchoalveolar lavage was negative for cytology.

Punch biopsy of one of the skin lesions demonstrated poorly circumscribed granulomas surrounding blood vessels and skin appendages, mild lymphocytic infiltration with no features to suggest cutaneous lymphoma, and no evidence of leukocytoclastic vasculitis. Stains for fungal and acid-fast bacilli were negative.

**Figure 2:**
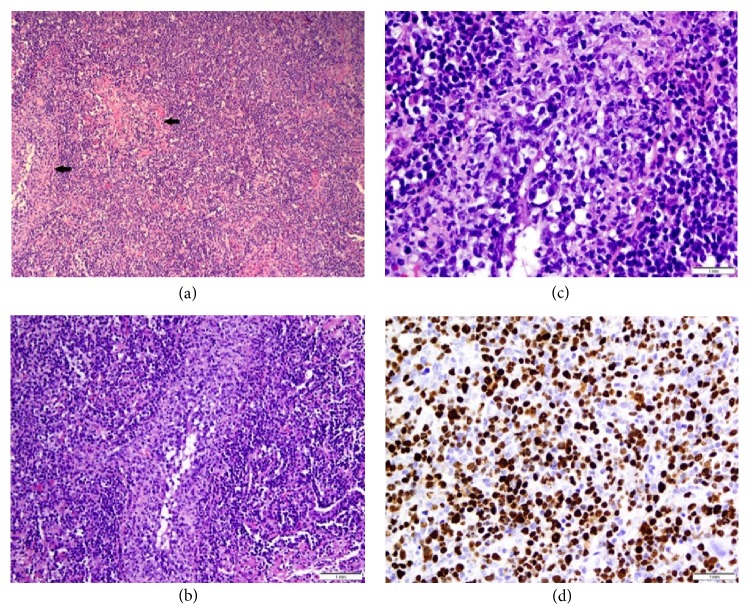
Low power section (a) from lung biopsy showing a diffuse neoplastic lymphoid cells (arrows) infiltrating the lymphatic and bronchovascular bundles. (H&E, 100x) Medium power view (b) showing neoplastic cells involving walls of pulmonary vessels. Small, reactive lymphocytes are seen in the background. (H&E, 200x) High power view (c) shows large, atypical B-cells with irregular nuclear membranes, open chromatin, and prominent nucleoli invading vessel walls (angioinvasion). (H&E, 500x) The neoplastic B-cells were positive for EBV (d).

**Figure 3:**
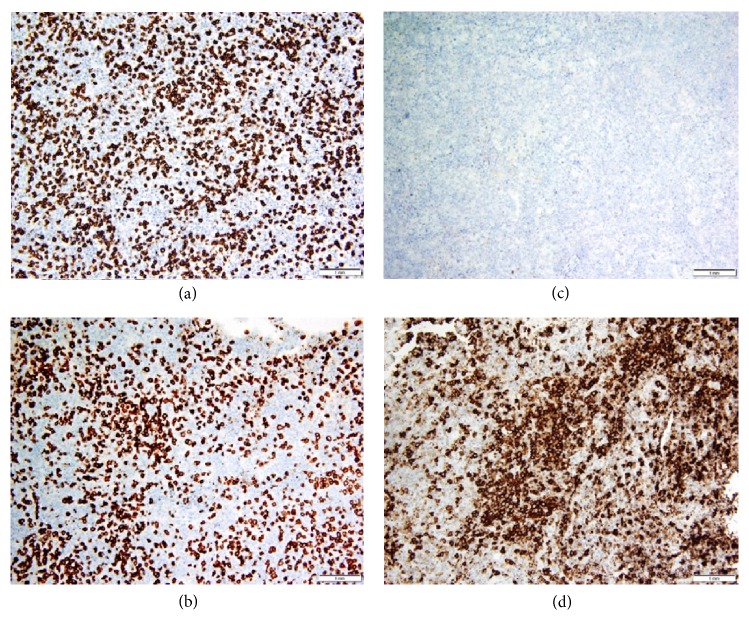
Immunostaining for CD3 (a) and CD5 (b) highlights background small, reactive T-cells. The large, atypical B-cells are negative. The large neoplastic B-cells are negative for CD5 and CD23 (c). CD20 is positive in large, atypical B-cells (d). The background small lymphocytes (reactive T-cells) are negative. The overall immunostaining pattern excludes both EBV+ CLL and EBV+ T-cell lymphoma as possible diagnoses.

Left upper and lower lobe wedge biopsies of the nodules were taken through video-assisted thoracoscopic surgery (VATS) as the diagnosis was still unclear. The biopsies revealed EBV-positive DLBCL with features of LG grade 3 (Figure 2). The features that favor LG over DLBCL include a background that consisted predominantly of inflammatory cells with a minority of large B-cells and vascular invasion. In addition, lung involvement and EBV positivity are not exclusive to LG, but are almost always present in LG [1]. Flow cytometry did not detect the large B-cells which are sometimes too fragile to survive flow cytometric processing, but did detect rare, small monoclonal B-cells with a CLL/small lymphocytic lymphoma (SLL) phenotype. The flow cytometric findings are compatible with the morphology as no significant CLL/SLL population could be identified with CD5 and CD23 immunostaining (Figure 3). In this case, the source of rare CLL cells is likely peripheral blood, either physiologically through the inflammatory response or contamination during resection. Features of secondary organizing pneumonia were also present.

**Figure 4:**
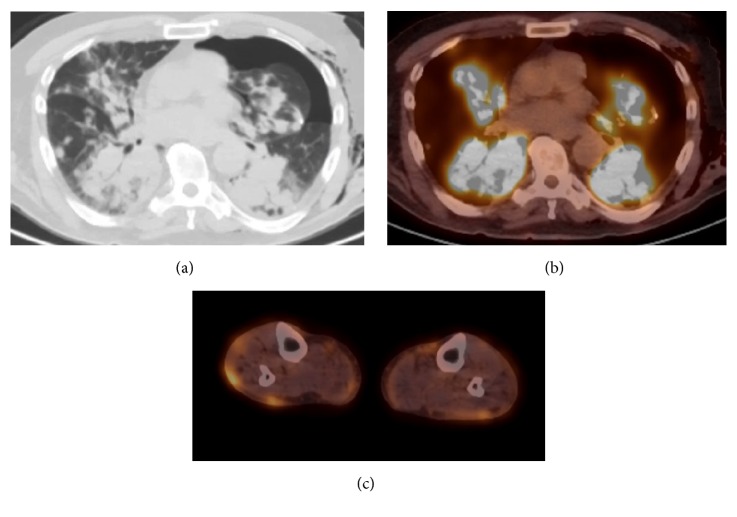
Axial images from PET/CT obtained 4 weeks following baseline chest CT. Axial CT image in lung windows (a) shows marked progression and confluence of bilateral peribronchovascular lung opacities. Fused PET/CT image of the thorax (b) shows the pulmonary abnormalities to have intense FDG uptake with max SUV of 22.3. There were no FDG-avid lymph nodes or splenomegaly. Left pneumothorax and subcutaneous emphysema were a result of the patient's recent VATS biopsy. Axial fused image of the lower extremities (c) shows FDG uptake within multiple subcutaneous nodules.

The patient underwent PET-CT 4 weeks following the initial chest CT (Figure 4). This showed significant progression and confluence of bilateral peribronchovascular lung opacities. The lung opacities had diffuse FDG uptake with a maximum SUV of 22.3. The PET/CT also demonstrated intense uptake in the cutaneous lesions of the lower extremities.

Bone marrow biopsy was consistent with hypercellular marrow with 20-30% marrow involvement by CLL/SLL cells. The patient was started on R-CHOP chemotherapy with Neupogen support. IVIG was also given for hypogammaglobulinemia.

## 2. Discussion

Lymphomatoid granulomatous (LG) is a rare EBV-associated B-cell lymphoproliferative disease with multiorgan involvement. It is most prevalent in men in the 4th through 6th decades [2]. The angiocentric and angiodestructive properties of lymphomatoid granulomatosis described by Liebow et al. are the hallmark of the disease [3]. LG can produce a spectrum of changes from inflammatory vasculitis to malignant lymphoma [4]. The diagnosis is made by a histologic triad of nodular polymorphic lymphoid infiltrates, angiitis, and granulomatosis [5]. The histologic grading scale ranges from 1-3 and is based on the degree of cytologic atypia and necrosis, and the number of EBV-positive cells correlates with the grade [2].

The lungs are affected in nearly all cases. Most patients will have symptoms of cough and dyspnea but can also have sputum production or hemoptysis. The predominant radiologic feature in LG is pulmonary nodules with irregular margins in a peribronchovascular distribution [6, 7]. The nodules may appear as diffuse infiltrates versus masses or areas of consolidation. Cavitation or necrosis can occur due to the angiodestructive properties [2]. A peripheral ground-glass halo and air-bronchograms have been reported with the pulmonary lesions, which were evident in our case. On PET-CT, the pulmonary lesions will be FDG-avid [8].

A pleural effusion may be present. Our case demonstrated nonenlarged mediastinal and axillary nodes, all of which were not FDG-avid and likely relate to underlying CLL. Lymph nodes, spleen, and bone marrow are not typically involved until later in the course of the disease, which differs from typical cases of lymphoma. Approximately 45% of individuals with LG have skin involvement such as patchy painful lesions or nodules that may ulcerate. CNS lesions are seen in 25-35% of cases and can present as a mass lesion or as various neurologic-type symptoms such as altered mental status or seizure [2]. Renal and liver involvement have been reported in autopsy cases although clinically significant disease is rare.

LG can be included in a long radiologic differential which often but not always includes pneumonia, malignant lymphoma, pseudolymphoma, lymphocytic interstitial pneumonia, metastasis, cryptogenic organizing pneumonia, granulomatosis with polyangiitis, and sarcoidosis. Wegener's granulomatosis is one of the mimickers of this diagnosis as it shares many features with LG including pulmonary nodules with cavitation or peripheral ground-glass halo, skin involvement, and renal involvement. The differential should also include other etiologies of cavitating lung nodules such as septic emboli. One may include sarcoidosis and Kaposi sarcoma in the differential given the peribronchovascular distribution of nodules.

Both CLL and LG are independently known to be associated with suppressed immunity, as evidenced in this case with hypogammaglobulinemia [9]. It is possible that the patient's history of CLL and immune suppression could be a risk factor for the development of LG.

CLL is known as an indolent type of lymphoma and should not demonstrate FDG activity. However, increased metabolic activity will occur in cases of Richter transformation, especially if the max SUV is greater than 5 [10]. The FDG negative mediastinal and axillary lymph nodes support the conclusion that the lymph nodes on CT are of CLL nature, as DLBCL of any subtype is FDG-avid.

Lung nodules have a very low chance of being malignant if there is no uptake. There is a positive predictive value for malignancy of 90% if the SUV is above 2.5, and 80% if SUV is between 1.5 and 2.0 [11]. The highly FDG-avid lung lesions in our case with a max SUV of 22.3 support an aggressive tumor such as DLBCL.

Whether LG represents Richter syndrome or not is debatable. A minority of patients with CLL (2-10%) will develop a more aggressive second lymphoid malignancy, including prolymphocytic leukemia, DLBCL, and Hodgkin lymphoma [12–14]. Classically, Richter syndrome is associated with DLBCL, likely because DLBCL is most common histologic transformation of CLL [15]. But, similar to DLBCL, LG is a more aggressive lymphoma composed of large B-cells. The distinction between DLBCL and LG lies in the latter's characteristic localization (lung in>90%), histologic features (angiodestructive lesions in an inflammatory background), and EBV positivity [1]. DLBCL can also show any one of these features, but not as consistently as LG.

It is tempting to employ molecular studies to establish a clonal relationship between the LG and CLL in this case; however this has not been a requirement for the diagnosis of Richter syndrome. In fact, one study showed that 22% of patients with Richter syndrome had DLBCL and CLL components that were clonally unrelated [16].

Molecular risk factors implicated in the transformation of CLL to RS include germline genetic characteristics (BCL-2 GG genotype, CD38 GG genotype, and LRP4 TT genotype), biologic characteristics of CLL (unmutated IGHV, stereotyped B-cell receptors, telomere length less than 5000 base pairs, and the positive expression of CD38, CD49d and ZAP-70), somatic genetic events (CDKN2A deletion, TP53 disruption, C-myc activation, trisomy 12, Notch-1 mutation, and heterogenous genomic aberrations), disease stage, and possibly the type of CLL therapy. It is unclear if any of these risk factors played a role in this case [17].

Comparison of immunoglobulin gene rearrangements was not performed in this patient; however this would have no effect on diagnosis or treatment, as secondary aggressive lymphomas in these patients are typically treated like de novo malignancies.

The ability to diagnose LG strictly from radiologic findings is unlikely. However, in the correct clinical setting and in an EBV-positive patient in particular with the correct radiographic findings, it is a differential which should be considered.
